# 2307. Respiratory Virus Testing Strategy on Admission to Hospital in a Season where SARS-CoV-2 is Co-circulating with Seasonal Respiratory Viruses

**DOI:** 10.1093/ofid/ofad500.1929

**Published:** 2023-11-27

**Authors:** Paige Reason, Victoria R Williams, Lorraine Maze Dit Mieusement, Heather Candon, Xena Li, Robert Kozak, Jerome A Leis

**Affiliations:** Sunnybrook Health Sciences Centre, Toronto, Ontario, Canada; Sunnybrook Health Sciences Centre, Toronto, Ontario, Canada; Sunnybrook Health Sciences Centre, Toronto, ON, Toronto, ON, Canada; Sunnybrook Health Sciences Centre, Toronto, Ontario, Canada; Shared Hospital Lab, Toronto, Ontario, Canada; Sunnybrook Health Sciences Centre, Toronto, Ontario, Canada; Sunnybrook Health Sciences Centre, Toronto, Ontario, Canada

## Abstract

**Background:**

Syndromic multiplex testing is the mainstay of viral respiratory surveillance, yet some evidence supports the use of asymptomatic testing for SARS-CoV-2 on patient admission. We conducted a prospective quality improvement study to determine the adherence and outcomes associated with a hybrid surveillance strategy for patients being admitted to an acute care hospital who underwent testing on admission.

**Methods:**

In November, 2022, corporate education recommended that patients with compatible viral respiratory symptoms undergo respiratory multiplex testing for 15 viral pathogens, while those without viral respiratory symptoms (considered asymptomatic) undergo SARS-CoV-2 PCR testing alone. Between December 1, 2022 to March 30, 2023, we measured the testing strategy using positivity rate, rate of omission (i.e., SARS-CoV-2 testing for symptomatic patients, defined as needing a repeat swab for multiplex within 24-hours of admission), and rate of commission (i.e., multiplex testing for asymptomatic patients). Those with detection of non-SARS-CoV-2 respiratory viruses in absence of symptoms were prospectively assessed for 24 hours for development of symptoms and roommate contacts underwent syndromic surveillance for 72-hours.

**Results:**

Among 5659 patients tested on admission, 2868(50.6%) underwent multiplex testing while 2791(49.3%) were tested only for SARS-CoV-2. The positivity rate for multiplex testing, positivity rate for COVID SARS-CoV-2 testing, omission rate and commission rate were 249/2868(8.7%), 81/2791 (2.9%), 259/1403(17.8%), 1618/4091(39.6%), respectively. Symptom status for 165 patients was not available. Asymptomatic non-SARS-CoV-2 positive patients made up 0.6% of all admissions, of whom none developed symptoms within 24 hours, and the attack rate among roommate contacts was zero (0/20). There were only 31 additional days of precautions for asymptomatic non-SARS-CoV-2 respiratory virus detection that made up 4.4% of overall precaution-days for respiratory virus infection.

**Conclusion:**

A hybrid surveillance strategy for patients admitted to hospital based on presence of symptoms was implemented with relatively low rates of repeat testing and asymptomatic non-SARS-CoV-2 virus detection.

**Disclosures:**

**Jerome A. Leis, MD MSc FRCPC**, Ontario Hospital Association, Ministry of Attorney General of Ontario, Seneca College: Expert Testimony

